# An Anatomical Variant of the Accessory Right Hepatic Artery Arising From the Superior Mesenteric Artery: A Cadaveric Case Report

**DOI:** 10.7759/cureus.115996

**Published:** 2026-09-09

**Authors:** Maha F Kirmani, Joshua Pound, Christine Abreo, Tiffany Kottukkal, Jack Davis, Mustafa Saed, John K Hubbard

**Affiliations:** 1 Medicine, Texas A&M College of Medicine, Bryan, USA; 2 Neuroscience and Experimental Therapeutics, Texas A&M College of Medicine, Bryan, USA

**Keywords:** accessory right hepatic artery, anatomical variation, hepatic artery, hepatobiliary anatomy, superior mesenteric artery

## Abstract

The right hepatic artery (RHA) provides blood flow to the right lobe of the liver. In typical anatomy, the RHA branches from the proper hepatic artery, which branches from the common hepatic artery originating from the celiac trunk of the abdominal aorta. During a standard cadaveric dissection, a large accessory right hepatic artery (aRHA) was noted arising directly from the superior mesenteric artery. The lumen of the identified aRHA measured approximately 1 cm in diameter. The artery also appeared darker in color than the surrounding vasculature, with no evidence of prior surgery or procedures. The size and position of this variant are important to note before surgery, given its large size and its significant role in supplying the liver. This highlights the importance of thorough preoperative vascular imaging to identify such anomalies and prevent inadvertent vascular injury.

## Introduction

The right hepatic artery (RHA) is a branch of the proper hepatic artery (PHA), which branches from the common hepatic artery, which arises from the celiac trunk of the aorta. The RHA supplies the right lobe of the liver with oxygenated blood and travels posterior to the common hepatic duct and cystic duct. The RHA, along with the portal vein and common bile duct, travels within the hepatoduodenal ligament [1].

There are a variety of known anatomical variants of the RHA that supply the liver and can influence planning for upper abdominal surgeries such as liver resection, pancreatoduodenectomy, and laparoscopic cholecystectomy. Approximately 80% of the population is thought to have the typical hepatic artery anatomy, in which the common hepatic artery gives rise to the proper hepatic artery, which then bifurcates into the left and right hepatic arteries supplying the respective lobes of the liver [1]. However, accessory right hepatic arteries (aRHAs) have been reported as anatomical variants occurring in 0.6-7% of subjects, with Kobayashi et al. further classifying rare hepatic artery variations [2]. Michels (1966) and Hiatt et al. (1994) described several branching patterns of hepatic arteries based on arterial origin and distribution [3,4].

In this case report of a human cadaver, a large aRHA was discovered supplying the right lobe of the liver. This study aimed to describe and report the anatomical variant of the noted RHA.

Anatomic variations in blood supply can play a significant role in upper abdominal surgeries, as potential anomalies can lead to further complications following a completed procedure. This includes hepatic artery aneurysms, which are one of the more common forms of visceral artery aneurysms [5]. Further exploration of variations in hepatic artery anatomy will be undertaken to clarify their implications. This study reports and explores the implications of this anatomical variant.

This article was previously presented as a poster at the 2023 American Association of Anatomy Regional meeting on October 7, 2023.

## Case presentation

During a first-year medical gross anatomy dissection laboratory course at Texas A&M University College of Medicine, a routine dissection of a 90-year-old female cadaver in the upper abdominal region revealed an accessory artery to the liver in addition to the usual left and right hepatic arteries. The variant artery was observed branching to the right and lateral aspect from the SMA and then entering superiorly into the right lobe of the liver. The vessel was sectioned, and the diameter of the lumen was measured with digital calipers (Qfun, IP54), which was found to be approximately 1 cm. Further observation of the cadaver was conducted, and it was determined that, grossly, there were no significant changes in the caliber of the surrounding vasculature and no anastomoses were present. Schematic representations were developed to demonstrate the anatomical variations observed in this case (Figure 1).

**Figure 1:**
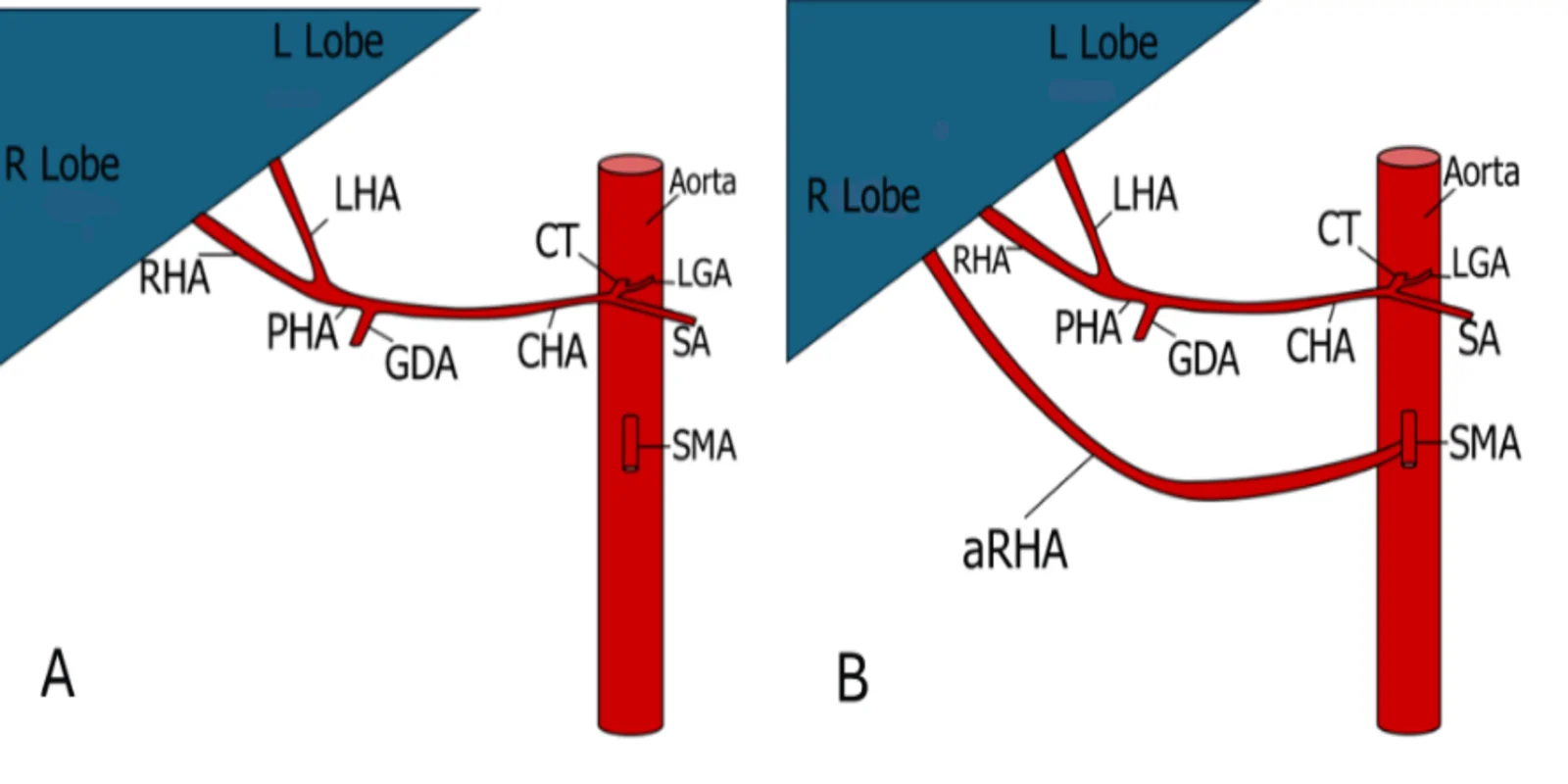
Typical anatomy vs. observed abnormal anatomy A. Typical anatomy; B. Observed anatomy with accessory right hepatic artery (aRHA) LGA (left gastric artery), SA (splenic artery), CT (celiac trunk), CHA (common hepatic artery), GDA (gastroduodenal artery), PHA (proper hepatic artery), LHA (left hepatic artery), RHA (right hepatic artery), SMA (superior mesenteric artery), aRHA (accessory right hepatic artery) This schematic was created by the authors using Microsoft PowerPoint (Microsoft Corporation, Redmond, WA, US).

Michels and Hiatt have described various hepatic arterial variations based on origin and branching pattern. Type I represents normal anatomy, while other types outline replaced or accessory arteries and variants. Michels classified the hepatic arterial variation based on the branching points and origin of the artery, while Hiatt et al. simplified the variations into a broader category [3,4]. The classification of these hepatic arterial variations is summarized in Table 1. Based on this table, it was determined that, as an aRHA, it followed the appearance of Michels VI and Hiatt III. In cases of aRHA, this variation can be derived from the superior mesenteric artery (SMA), which is responsible for supplying the duodenum, cecum, ascending colon, and the proximal one-third of the transverse colon [6].

**Table 1:** Original summary created by the authors based on the hepatic artery classification systems proposed by Michels (1966) and Hiatt et al. (1994) Sources: [3,4]

Description	Michels Type	Hiatt Type
Normal anatomy	I	I
Replaced RHA from SMA	III	III
Replaced RHA and LHA	IV	IV
Accessory RHA	VI	III
CHA from SMA	IX	V

Based on its course, this artery was identified as an aRHA, as noted in Figure 1B. Also of note, the aRHA was darker in color compared to the surrounding vasculature (Figures 2, 3), with an abrupt change in color confined to the aRHA, where it branched from the SMA. This color change was strictly localized to the aRHA and originated from the SMA. Based on close study, we asserted that a generalized fixative effect was unlikely to be the cause of the local discoloration. However, since histological and biochemical studies were not performed, the cause could not be established clearly. There were no indications of sutures or past procedures that would implicate a possible cause for this discoloration. The cause of death (cerebrovascular atherosclerosis with thrombosis) did not directly pertain to the observed variation. No further medical history was available.

**Figure 2:**
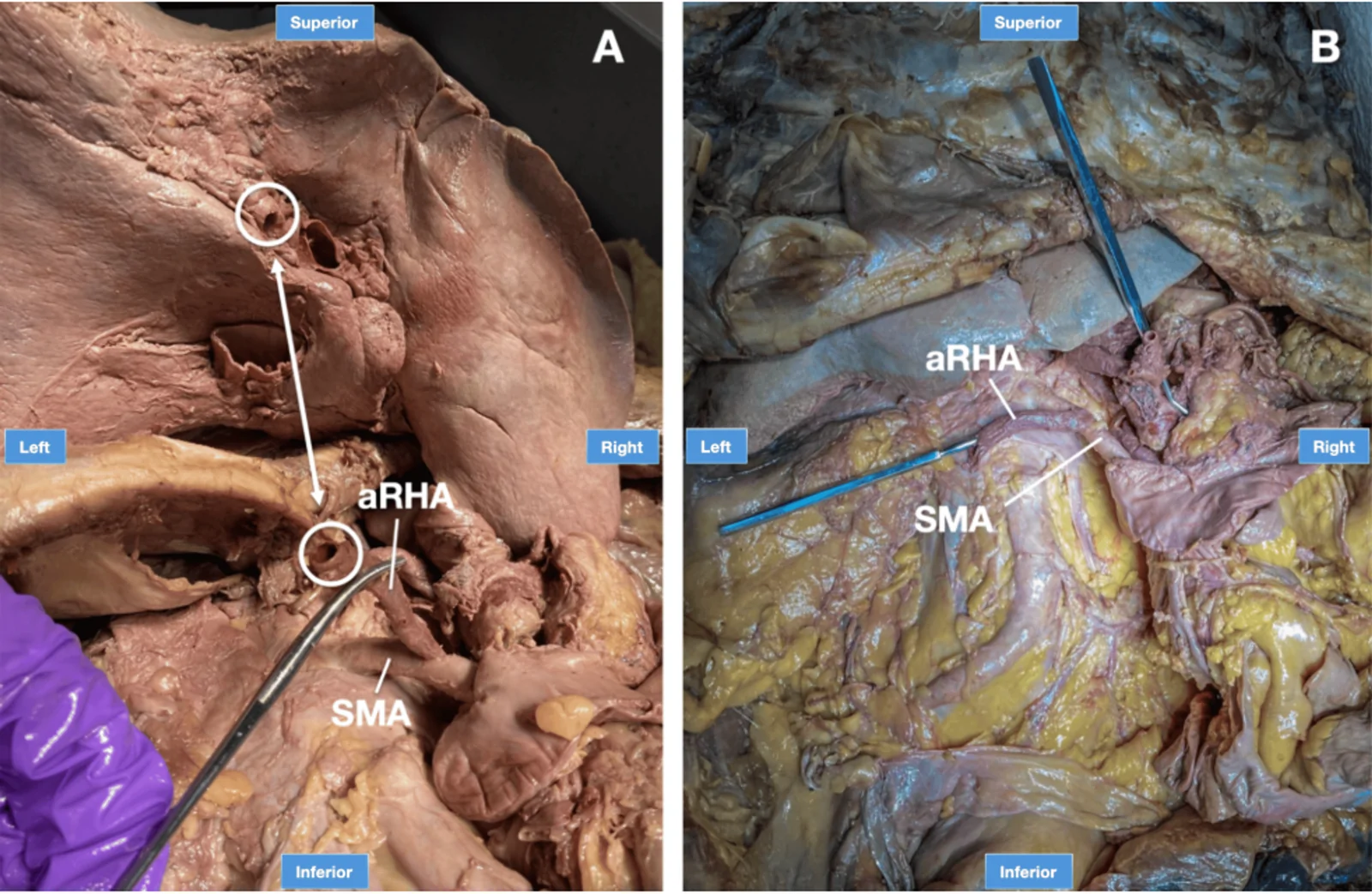
Cadaveric images of the aRHA arising from the SMA A. The accessory right hepatic artery (aRHA) and its entry into the liver. B. Zoomed-out view of the aRHA branching from the superior mesenteric artery (SMA) and overlying vasculature.

**Figure 3:**
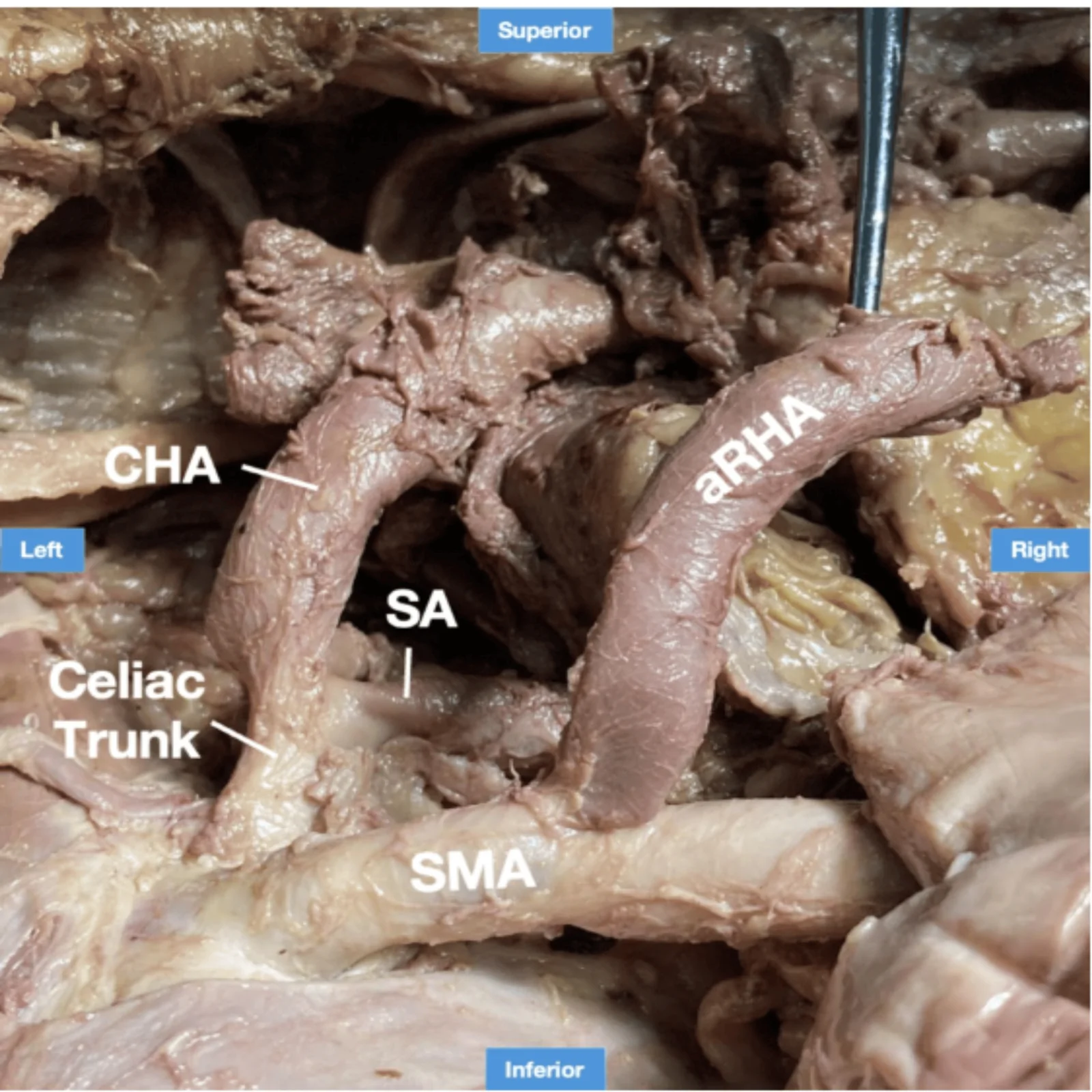
Diameter and noted discoloration of the aRHA Closer view of the 1 cm luminal diameter and noted discoloration of the accessory right hepatic artery (aRHA) derived from the superior mesenteric artery (SMA). Note that the common hepatic artery (CHA) is in proximity and not connected to the aRHA. The celiac trunk and splenic artery (SA) are also visible in the image and are located in close proximity to the aRHA.

A 1 cm accessory aRHA could cause significant complications during surgical procedures if not identified beforehand. Of all structures observed and dissected in this region, only the aRHA was of an atypical size [7]. No other medical conditions were noted, nor were any other anatomic variations in the abdominal vasculature.

## Discussion

aRHA variations can impact surgical operations, such as liver transplantation, cholecystectomy, and pancreatic surgeries, to avoid accidental lacerations leading to liver necrosis or hypovolemic shock. With the liver receiving 25% of cardiac output, equating to 800-1,200 mL/min, inadvertent laceration of an aRHA could lead to hypovolemic shock and hemodynamic instability [8]. With the 1 cm size of this aRHA, the implications of not considering this during surgical procedures are life-threatening to the patient.

Based on a review of 19,013 variants of the hepatic artery, an aRHA was found in 1.6% of cases [9]. Referencing Table 1, our specific finding can be classified as Michels VI and Hiatt III. This classification has clinical implications for patients, especially when considering the context of hepatobiliary surgeries. The majority of isolated hepatic artery (arteries supplying the liver and other neighboring structures) injuries heal without incident, yet about 11-76% of patients with bile duct trauma and RHA damage have reported ischemic liver damage following a hepatic artery injury. In cases with pancreatic fistula, hepatic artery (HA) adventitia is further damaged by excessive manipulation and compression. Clinical consequences of an HA injury include liver abscess, bile duct stenosis, and right lobe atrophy [10]. There is a priority to preserve the structures in hepatobiliary pancreatic surgeries so that the vascular supply is not disrupted. In the case of aberrant arterial structures, such as the one discovered in the dissection lab, this artery has a close relationship with the head of the pancreas. This structure is more vulnerable to damage when performing pancreatic head resections. More severe consequences linked to aRHA damage in surgery include insufficient hepatic volume, hepatic artery thrombosis, and ischemic cholangiopathy [11]. To avoid these complications, the use of arteriography and enhanced CT scans can help visualize the arterial vasculature, guiding surgical planning. By employing these imaging techniques preoperatively, surgeons can potentially minimize the risk of severe complications associated with hepatic artery variations.

The limitations of this case report include the lack of measurements between the anatomical variant and adjacent structures, including the hepatoduodenal ligament and bile duct. Additionally, determining the branching pattern of the aRHA and its course to specific hepatic lobes was challenging.

## Conclusions

This case of a rare, large accessory right hepatic artery (aRHA) arising from the superior mesenteric artery (SMA) highlights the importance of recognizing anatomic anomalies and their potential consequences if overlooked. There are also only a few reports describing this specific vascular variant, limiting opportunities for comparison with previously documented cases. Future studies will need to address further characterization of the aRHA variants through systematic anatomical and radiological approaches. Further understanding of the anatomic variations and clinical implications can be achieved with measurements that outline the artery's relationship to nearby structures such as the hepatoduodenal ligament, bile duct, and portal vein. Awareness of hepatic arterial variants, along with careful preoperative evaluation using advanced imaging modalities, including computed tomography and arteriography, is essential for safe surgical planning. Early identification and preservation of aberrant arterial anatomy may help minimize intraoperative risk and improve patient outcomes.
